# Single-Cell Transcriptomics of Human Tonsils Reveals Nicotine Enhances HIV-1-Induced NLRP3 Inflammasome and Mitochondrial Activation

**DOI:** 10.3390/v16111797

**Published:** 2024-11-20

**Authors:** Nadine Schrode, Trinisia Fortune, Aislinn M. Keane, Jesse F. Mangold, Benjamin Tweel, Kristin G. Beaumont, Talia H. Swartz

**Affiliations:** 1 Department of Genetics and Genomic Sciences, Icahn School of Medicine at Mount Sinai, New York, NY 10029, USA; nadine.schrode@mssm.edu (N.S.); kristin.beaumont@mssm.edu (K.G.B.); 2 Division of Infectious Diseases, Department of Medicine, Icahn School of Medicine at Mount Sinai, New York, NY 10029, USA; trinisia.fortune@mssm.edu; 3 Graduate School of Biomedical Sciences, Division of Infectious Diseases, Department of Medicine, Icahn School of Medicine at Mount Sinai, New York, NY 10029, USA; aislinn.keane@icahn.mssm.edu; 4 Medical Scientist Training Program, Graduate School of Biomedical Sciences, Icahn School of Medicine at Mount Sinai, New York, NY 10029, USA; 5 Department of Otolaryngology, Icahn School of Medicine at Mount Sinai, New York, NY 10029, USA

**Keywords:** HIV-1, nicotine, NLRP3 inflammasome, oxidative stress, mitochondrial dysfunction

## Abstract

Background: HIV-1 infection, even with effective antiretroviral therapy (ART), is associated with chronic inflammation and immune dysfunction, contributing to long-term health complications. Nicotine use, prevalent among people with HIV (PWH), is known to exacerbate immune activation and disease progression, but the precise biological mechanisms remain to be fully understood. This study sought to uncover the synergistic effects of HIV-1 infection and nicotine on immune cell function, focusing on beneficial insights into NLRP3 inflammasome activation, oxidative stress, and mitochondrial pathways. Methods: Human tonsil explants were infected with HIV-1 and exposed to nicotine. Single-cell RNA sequencing was used to profile immune cell populations and gene expression linked to inflammasome activation, oxidative stress, and mitochondrial function. Gene set enrichment analysis (GSEA) and synergy assessments were conducted to investigate how nicotine modulates immune responses in the context of HIV. Results: The combination of HIV infection and nicotine exposure significantly increased NLRP3 inflammasome activation, thioredoxin, and components of oxidative phosphorylation. Conclusions: This study highlights how the combined effects of HIV-1 and nicotine offer valuable insights into immune modulation, opening doors for future therapeutic strategies. Targeting the NLRP3 inflammasome and addressing nicotine use may contribute to improved outcomes for PWH.

## 1. Introduction

HIV-1 infection remains a global health challenge, affecting millions of individuals worldwide. While antiretroviral therapy (ART) has significantly improved the prognosis for people with HIV (PWH) by suppressing viral replication, chronic inflammation and immune dysregulation persist, contributing to comorbidities such as cardiovascular disease, neurocognitive disorders, and premature aging [1,2,3,4,5,6,7]. Among the factors exacerbating these complications, nicotine use—through smoking or other means—has emerged as a critical contributor to disease progression and inflammation in PWH [8,9,10,11,12,13].

Nicotine, a known pro-inflammatory agent, causes direct damage to the immune system and induces oxidative stress and metabolic dysregulation [8,9]. These processes can potentiate HIV pathogenesis, leading to higher viral reservoirs, increased immune activation, and poorer clinical outcomes. Smoking rates remain disproportionately high among PWH compared to the public, making nicotine a relevant co-factor in the management of HIV infection [9,13,14,15,16,17]. Notably, nicotine’s inflammatory effects may extend to the central nervous system, where it exacerbates neuroinflammation, blood–brain barrier dysfunction, and oxidative stress—factors that contribute to HIV-associated neurocognitive disorders (HAND) [11,18,19]. The impact of nicotine on HIV-induced inflammation and the mechanisms underlying this interaction, however, remain incompletely understood.

Recent studies have identified the NLRP3 inflammasome as a key player in chronic inflammation in HIV infection [20,21,22,23,24,25,26,27,28,29,30,31,32,33,34,35,36]. The inflammasome, a multiprotein complex that activates caspase-1 and triggers the release of pro-inflammatory cytokines such as IL-1β, has been implicated in HIV persistence and immune dysfunction. Notably, nicotine has been shown to activate the NLRP3 inflammasome further, raising the question of whether nicotine exacerbates HIV-induced inflammation through this pathway [37,38,39,40]. Our laboratory has demonstrated that the HIV-1 infection of CD4^+^ T cells upregulates genes associated with oxidative phosphorylation, leading to increased metabolic activity and immune activation [36]. Additionally, macrophages in proximity to infected T cells exhibit elevated expression of NLRP3 inflammasome components, suggesting a localized interaction that amplifies inflammation within lymphoid tissue, specifically in tonsils. This tissue is highly relevant as a viral reservoir site and offers a representative microenvironment to study the immune response and inflammatory signaling induced by HIV and exacerbated by nicotine.

In this study, we investigated the combined effects of HIV infection and nicotine exposure on immune cell populations, focusing on the role of inflammasome activation, oxidative stress, and mitochondrial dysfunction. We used ex vivo human tonsil explants and single-cell RNA sequencing to explore how nicotine impacts HIV infection and immune responses at the cellular and molecular levels. By analyzing key inflammatory and metabolic pathways, we aimed to elucidate the mechanisms driving the synergistic effects of HIV and nicotine and to identify potential therapeutic targets for reducing inflammation and improving outcomes in PWH who smoke.

Our findings demonstrate that HIV infection and nicotine exposure synergistically activate the NLRP3 inflammasome and induce oxidative stress in both CD4^+^ T cells and myeloid cells, contributing to increased inflammation and viral persistence. These insights provide a basis for novel therapeutic strategies, including inflammasome inhibition and nicotine cessation, to reduce chronic inflammation, limit viral reservoirs, and improve the health of PWH.

## 2. Materials and Methods

**Plasmids and viruses**. Plasmids expressing HIV-1 Env of 6535 (clone 3 (SVPB5)), BaL (clone BaL.01), REJO (pREJO4541 clone 67 (SVPB16)), and ZM109 (ZM109F.PB4, SVPC13) were used for generating HIV-1 pseudoviruses. These plasmids were obtained from Drs. David Montefiori, Feng Gao, Ming Li, John Mascola, B.H. Hahn, J.F. Salazar-Gonzalez, C.A. Derdeyn, and E. Hunter through the NIH ARP. The plasmid bearing SF162 (pIRESSF162) env was constructed as previously described [41,42]. HIV-1 NL-CI contains mCherry in place of nef, and nef expression is directed by a downstream internal ribosome entry site (IRES) [43]. PBMCs were infected with NL-CI, which contains the NL4–3 envelope (X4 tropic), or RHPA, which was constructed by insertion of the R5-tropic B-clade primary envelope from pRHPA4259 clone 7 (SVPB14) into NL-CI [44]. The gene for the RHPA clone was obtained from B. H. Hahn and J. F. Salazar-Gonzalez (ARP). HIV-1 Gag-iCre is an HIV-1 clone carrying Cre recombinase as a Gag-internal gene fusion that releases active Cre into cells upon viral entry activating a recombinatorial gene switch changing dsRed to GFP-expression, as previously described [45]. NL-CIΔCT was cloned by generating a PCR fragment of the C-terminal Env from the NL-CIΔCT plasmid, as previously described [46]. Production of pseudoviruses was accomplished through co-transfection of 293T/17 cells with HIV-1 rev- and env-expressing plasmids and the pNL4–3Δenv R-E- plasmid using the jetPEI transfection reagent (Polyplus-transfect SA, Illkirch, France). After 48 h, harvested supernatants were clarified by high-speed centrifugation (Sorvall ST 40R Centrifuge, Thermo Fisher Scientific, Madison, WI, USA) at 100,000× *g* at 4 °C for 2 h and 0.45 μm filtration. Viral stocks were quantified by the HIV-1 p24 antigen via enzyme-linked immunosorbent assay (ELISA) with coating antibody D7320 and sheep anti-HIV-1-p24 gag (Aalto Bio Reagents, Dublin, Ireland), as described previously [46]. Single-use aliquots were stored at −80 °C.

**Flow cytometry and gating strategy**. An Attune NxT Flow Cytometer (Thermo Fisher Scientific) was used to detect infection and discriminate donor and target cell populations. Viable cells in productive infection assays were detected with LIVE/DEAD Fixable Dead Cell Stain (Life Technologies, Carlsbad, CA, USA), an amine-reactive fluorescent dye that can penetrate the membranes of dead cells but not live cells. Samples were stained with LIVE/DEAD Fixable Violet Dead Cell Stain at a concentration of 1:1000 in Wash Buffer (PBS supplemented with 2 mM EDTA and 0.5% bovine serum albumin). Stained cells were incubated at 4 °C for 30 min, then were washed and fixed in 2% paraformaldehyde for flow cytometry. All cells were initially discriminated by side scatter (SSC) area versus forward scatter (FSC) area (SSC-A/FSC-A); doublets were excluded using SSC-A vs. SSC-H and dead cells were excluded by gating on the negative populations for LIVE/DEAD Fixable Dead Cell Stain. In productive infection assays, infection was detected by the presence of mCherry in cells infected with HIV-1 NL-CI, dsRed-Express and mCherry were detected using the phycoerythrin-Texas Red (PE-Texas Red) channel, LIVE/DEAD Fixable Violet Dead Cell Stain was detected with the 3-carboxy-6,8-difluoro-7-hydroxycoumarin (Pacific Blue, Melbourne, Australia) channel, and LIVE/DEAD Fixable Blue Dead Cell Stain was detected with the 4′,6-diamidino-2-phenylindole (DAPI) channel. In HIV-1 fusion assays, donor cells labeled with eFluor 450 were detected in Alexa Fluor 405-A channel, and target cells that expressed dsRed were detected using Alexa Fluor 568-A channel. All cells within a single experiment were analyzed using the same instrument settings.

**Productive infection assays**. Target MT-4 cells were infected in 96-well plates with HIV-1 NL-CI to obtain up to 10% infection after 48 h in the absence of inhibitors. MT-4 cells were pre-incubated with antagonists for 30 min at 37 °C before infection with HIV-1. At 48 h after infection, cells were fixed in 2% paraformaldehyde, and infection was quantified via mCherry fluorescence in flow cytometry. For the time-of-addition assay, P2X1 antagonists were added to MT-4 cells at the indicated time points of HPI with HIV-1 NL-CI (1.62 ng p24 per well), as previously described [47]. At 48 h after mixing, cells were stained and fixed in 2% paraformaldehyde for flow cytometry, as described above. For coreceptor competition assays, MT-4 cells were co-incubated with nicotine for 30 min at 37 °C before infection with HIV-1 NL-CI (1.62 ng p24 per well). At 48 h post-infection, cells were stained and fixed in 2% paraformaldehyde for analysis via flow cytometry as described above.

**Preparation and ex vivo infection of human tonsil explant tissue blocks.** Human tonsils were collected within several hours of routine tonsillectomies performed by B. Tweel at the Mount Sinai Health System in New York City under an Institutional Review Board-approved protocol. Human tonsil explants were dissected into 2 mm tissue blocks that were plated on top of collagen sponge (GelFoam; Pfizer, New York, NY) and maintained in RPMI 1640 medium (Life Technologies) containing 15% fetal bovine serum (FBS; Sigma, St. Louis, MO, USA), 2 mM GlutaMax (Life Technologies), 2 mM L-glutamine (Corning, New York, NY, USA), 1 mM sodium pyruvate (Corning), 1% minimal essential medium (MEM) nonessential amino acids (Corning), 2.5 μg/mL amphotericin B (HyClone, Logan, UT), 50 mg/mL gentamicin sulfate (Corning), and 0.3 mg/mL Timentin (bioWORLD, Dublin, OH, USA) as previously described [48,49]. The human tonsil explant tissue blocks were left uninfected or individually inoculated with 4 mL of HIV-1 NL-CI (equivalent to 3.24 ng of p24) in the presence or absence of nicotine. Supernatants were exchanged every 2 to 3 days, aliquoted, and stored at −80 °C. HIV-1 p24 antigen concentration in the supernatant was quantified using a b-galactosidase-based luciferase assay (Promega, Madison, WI, USA) with TZM-bl target cells as previously described [44,49].

**Single-cell RNA sequencing**. We used the 10X Chromium Single-Cell Gene Expression approach to characterize gene expression patterns from infected cells, bystanders of infected cells, and uninfected cells within the complex mixture of HIV-infected tonsillar cells. Fresh tissues were dissociated into single-cell suspensions using the GentleMACS Octodissociator with kit matched to the tissue type (Miltenyi Biotech, Auburn, CA), following manufacturer’s instructions. Viability of single cells was assessed using Trypan Blue staining, and debris-free suspensions of >80% viability were deemed suitable for single-cell RNA Seq. Single-cell RNA Seq was performed on these samples using the Chromium platform (10x Genomics, Pleasanton, CA, USA) with the 3′ gene expression (3′ GEX) V3 kit, using an input of ~10,000 cells per sample. Briefly, gel beads in emulsions (GEMs) were generated on the sample chip in the Chromium controller. Barcoded cDNA was extracted from the GEMs by Post-GEM RT-cleanup and amplified for 12 cycles. Amplified cDNA was fragmented and subjected to end-repair, poly A-tailing, adapter ligation, and 10X-specific sample indexing following the manufacturer’s protocol. Libraries were quantified using Bioanalyzer (Agilent, Santa Clara, CA) and QuBit (Thermofisher, Madison, WI, USA) analysis. Libraries were sequenced in paired-end mode on a NovaSeq instrument (Illumina, San Diego, CA, USA) targeting a depth of 50,000–100,000 reads per cell. Sequencing data were aligned and quantified using the Cell Ranger Single-Cell Software Suite (version 3.0, 10x Genomics) against a custom GRCh38 human reference genome that includes the HIV_CL-NI viral sequence to identify cells expressing these transcripts.

**Data preprocessing**. The R-based package, Seurat (version 3.1.1) [50] was used to process the single-cell RNA sequencing data. Genes detected in <3 cells were filtered out of the analysis. Cells with expression of <500 total molecules, <200 or >2500 unique genes, >20% mitochondrial genes, and unique genes to total molecules in a ratio less than 2.2 were filtered out of the analysis. The datasets of each treatment group (control, HIV-infected, and HIV-infected and nicotine-treated) were merged and integrated. A total of 16,541 cells and 16,126 genes from 3 samples were included for further analysis.

**Quality control and sample integration**. Initial analysis began with the combination of all data sets. Subsequently, quality control (QC) metrics were applied to evaluate the relationships between read counts, transcript counts, and mitochondrial gene ratios. Read and transcript counts were expected to be roughly correlated, and mitochondrial gene expression was limited to below 20%. Cells identified as outliers, including those with a log10(genes)/log10(UMI) ratio below 0.8, or genes expressed in fewer than 0.5% of cells, were excluded from further analysis. This filtering ensured the retention of high-quality data for downstream analyses. Following QC, multiple integration approaches were applied to the datasets to address potential sample, donor, or batch effects. Known cell-type-specific markers and metadata were visualized to assess the suitability of each integration method. As a result, Harmony Integration was chosen, which iteratively adjusts for dataset-specific effects by performing fuzzy clustering in PCA-reduced space. Global and dataset-specific centroids are calculated within each cluster, and cell-specific corrections were applied to maximize cluster diversity. Harmony continues these adjustments through successive iterations until dataset dependencies are minimized, providing an optimal integrated dataset for downstream analysis. The integrated data object was used for all subsequent analyses, including differential expression, marker identification, and downstream functional assays.

**Identification of major cell clusters**. The SCTransform function [51] in the Seurat program was utilized to perform normalization, scaling, and variance stabilization of the data, as well as to regress out of the effects of the total number of molecules and the mitochondrial gene expression percentage. Dimensionality reduction was performed using principal component analysis (PCA) of the most variable 2000 genes. The first 40 principal components and a resolution of 1.0 were used for uniform manifold approximation and projection (UMAP) and clustering. Cell types were assigned using established canonical markers and automated prediction methods. A random forest classifier, trained using external data from Massoni-Badosa et al. [52], was applied to the current dataset, allowing for the assignment of cell types based on annotations from the reference data. Established canonical cell-type-specific markers were used to confirm cell-type annotations.

**Differential expression and pathway analysis.** Differential expression within each cluster between different conditions was performed using the FindMarkers function (Wilcoxon Rank Sum test) in Seurat. Gene set enrichment analysis (GSEA) and over-representation analysis (ORA) were performed using the GSEA and enricher functions in the clusterProfiler R package [53] in conjunction with the Reactome database [54].

**Synergy analysis**. To explore the observation of an apparent synergistic effect between HIV infection and nicotine treatment, we adapted a previously established protocol [55,56]. Briefly, the expected additive effect of differential expression in HIV infection and Nicotine treatment was modeled by adding the individual coefficient comparisons: (HIV-infected—unexposed) + (nicotine-treated—untreated). The synergistic effect was modeled by subtracting this additive effect from the combinatorial perturbation comparison: (HIV-infected and nicotine-treated—unexposed and untreated)—((HIV-infected—unexposed) + (nicotine-treated—untreated)). We categorized all genes by the direction of their change in both models and their log_2_(fold change) in the synergistic model. log_2_(fold change) standard deviations (SD) were calculated for all samples, and the mean was used as a baseline. Genes were grouped into ‘positive synergy’ if their fold change was greater than mean(SD) and ‘negative synergy’ if it was smaller. If the corresponding additive model log2(fold change) showed the same or no direction, the gene was classified as ‘more’ differentially expressed in the combinatorial perturbation than predicted.

**Data and code availability**. All relevant data are available from the authors and/or are included in the manuscript. Single-cell RNA sequencing data will be deposited to GEO. Software and codes used in the data analysis are either publicly available from the indicated references in the Section 2 or available upon request.

**Statistical analysis and calculations**. Comparisons were performed using GraphPad Prism 7, version 7.0d (GraphPad Software). DMSO-treated controls were set to 100%, and drug-treated conditions were expressed as a percentage of control. Statistical analyses were performed on inhibition data that reached ≥50% with a one-tailed Student’s *t*-test. A *p*-value of less than 0.05 was considered statistically significant.

## 3. Results

### 3.1. Nicotine Increases HIV-1 Infection and Alters Immune Cell Profiling 

**Figure 1:**
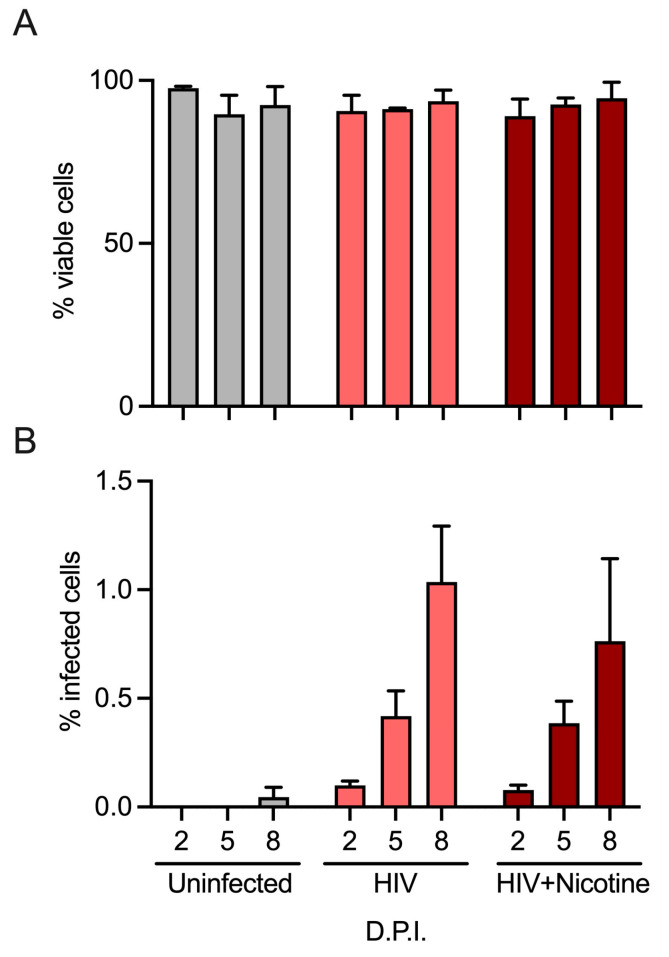
Infection of human tonsil explants by X4-tropic HIV-1_NL-CI_ treated with nicotine. Human tonsil explants were cultured on collagen rafts and infected with HIV-1_NL-CI_ or vehicle media. Supernatants were collected on days 2, 5, and 8 post-infection. Sloughed-off cells and media were separated by centrifugation, and a full media change was performed at each indicated time cells were collected. Cells were subjected to LIVE/DEAD staining and flow cytometry to evaluate viability (**A**). Suspension cells in supernatants were collected on day 8 for single-cell dissociation and processing for single-cell sequencing. Flow cytometry results are quantified as percent infection and percent viability in tonsils unexposed or exposed to HIV-1 (**B**). Mean values ± standard errors of the means from three to five donors.

First, the combined effects of nicotine and HIV-1 on infection levels and cell viability were evaluated. As shown in Figure 1, the effects of nicotine on HIV-1 infection and cell viability in human tonsil explants were evaluated over time. Cell viability was assessed across multiple conditions, including uninfected controls, HIV-infected samples, and treatment with nicotine (1 µM) (Figure 1A). Viability remained consistently high (>80%) throughout the experiment, regardless of the treatment condition or time point (days 2, 5, and 8 post-infection). There were no statistically significant differences in viability between HIV-infected and uninfected conditions, nor did nicotine treatment impact cell survival. The percentage of HIV-infected cells was measured at days 2, 5, and 8 post-infection (D.P.I.) (Figure 1B). HIV infection significantly increased over time, with the highest infection levels observed on day 8 (approximately 1% of cells). Nicotine treatment (1 µM) did not impact the percentage of infected cells.

**Figure 2:**
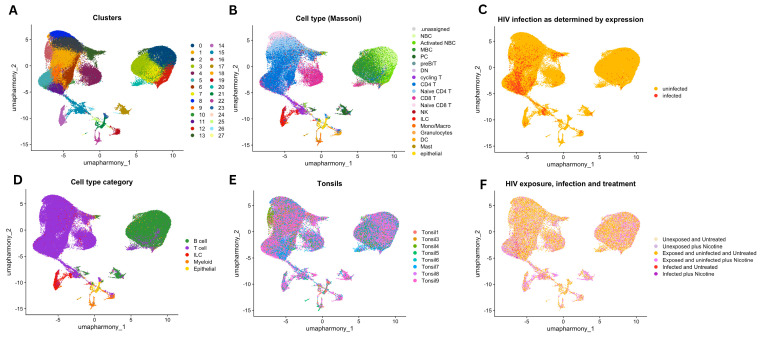
UMAP projections of single-cell RNA sequencing data from human tonsil explants. (**A**) Cells are color-coded by clusters identified through unsupervised clustering. (**B**) Cells are annotated with specific immune cell types based on marker expression from the Massoni reference dataset. (**C**) Cells are labeled as either infected or uninfected with HIV-1. (**D**) Cells are categorized into broader cell types, including B cells, T cells, ILC, myeloid, and epithelial cells. (**E**) Cells are color-coded by tonsil donors, representing data from nine individual donors. (**F**) Cells are stratified by HIV exposure, infection, and nicotine treatment status.

**Single-cell RNA sequencing was used to profile immune cell populations.** To gain deeper insights into the cellular responses driving this enhanced infection, we employed single-cell RNA sequencing to profile the tonsil explants under various conditions, as detailed in Figure 2. This high-resolution analysis enabled the identification of specific immune cell types and their involvement in HIV infection and nicotine exposure. Single-cell RNA sequencing data from human tonsil explants were visualized using UMAP projections to explore cell clusters, cell types, and the effects of HIV infection and nicotine treatment. Unsupervised clustering identified multiple distinct cell populations, with each cluster represented by a unique color (Figure 2A). These clusters reveal the heterogeneity of the tonsil explants, capturing a variety of cell populations present in the tissue. Cells were annotated with specific immune cell types based on known marker gene expression using the Massoni-Badosa reference dataset (Figure 2B). The annotated cell types include B cells, T cells, myeloid cells, and innate lymphoid cells (ILCs), among others. This annotation enabled the identification of functionally distinct cell populations within the tonsil explants. The cells were labeled by their infection status (infected or uninfected with HIV-1) (Figure 2C). HIV-infected cells are shown in orange, while uninfected cells are shown in yellow, highlighting the distribution of infection within the tissue. The UMAP plot demonstrates that HIV infection is dispersed across multiple cell types, suggesting that various immune cells are susceptible to infection. The cells are categorized into broader immune cell groups, including B cells, T cells, ILCs, myeloid cells, and epithelial cells (Figure 2D). Cells are grouped based on their donor origin, with data representing individual tonsil donors (Figure 2E). This allows the visualization of inter-donor variability in cell populations and highlights how different donors contribute to the overall dataset. Cells are indicated based on HIV exposure, HIV infection, and nicotine treatment status (Figure 2F). Together, these UMAP projections provide a comprehensive view of the immune cell landscape in human tonsil explants, showing the effects of HIV infection and nicotine treatment across various cell types and donor backgrounds.

### 3.2. HIV and Nicotine Co-Exposure Amplifies Inflammasome Activation and Immune Responses

**Figure 3:**
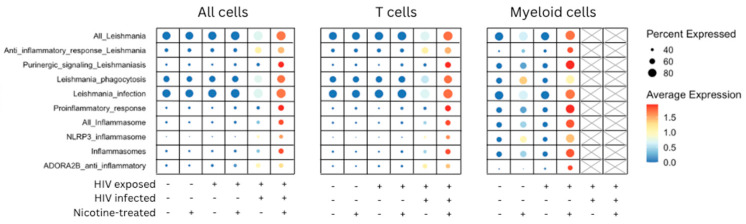
Expression dot plot representation of inflammasome pathway enrichment across all cells, T cells, and myeloid cells under HIV exposure, infection, and nicotine treatment. Expression of inflammasome-related genes across three cell populations—all cells, T cells, and myeloid cells—under various experimental conditions, including HIV exposure, HIV infection, and nicotine treatment. The *y*-axis lists inflammasome genes organized into relevant gene sets. The *x*-axis represents the combinations of experimental conditions indicated as either present (+) or absent (−) for HIV exposure, HIV infection, and nicotine treatment. The final two columns for myeloid cells are shaded to indicate that no infected myeloid cells were observed in these conditions. Dot size correlates with the abundance of gene expression within each population, while color intensity signifies the magnitude of expression.

Building upon the above characterization, Figure 3 demonstrates the expression of inflammasome-related genes. This figure examines how HIV exposure, infection, and nicotine treatment modulate the immune response at the gene expression level by analyzing key inflammasome gene sets across three distinct cell populations—all cells, T cells, and myeloid cells. It highlights the differential expression of inflammasome-related genes under these conditions. Across all cells, a distinct pattern of inflammasome gene expression was observed. AIM2 inflammasome and Leishmania pathways show high expression levels, particularly in conditions where cells were HIV-infected or exposed to nicotine. The color intensity, representing the average expression level, was strongest in these conditions, with the largest dots indicating a higher percentage of cells expressing these genes. The expression of inflammasome genes was similarly enriched in HIV-infected and nicotine-treated T cells. Notably, pathways such as NLPR3 inflammasome and pro-inflammatory response were significantly upregulated in these conditions. The combinatorial effect of HIV infection and nicotine treatment led to pronounced expression of inflammasome pathways in T cells. In myeloid cells, the expression patterns differed slightly, with a broader distribution of inflammasome-related genes. Although Leishmania pathways and pro-inflammatory response genes were still expressed, their expression levels were relatively lower than T cells and all cells, as shown by the lighter color and smaller dot size. Nevertheless, nicotine treatment had a distinct effect on increasing gene expression in the AIM2 inflammasome and ADORA2B anti-inflammatory pathways in myeloid cells.

### 3.3. Nicotine and HIV Enhance Oxidative Stress and Mitochondrial Dysfunction

**Figure 4:**
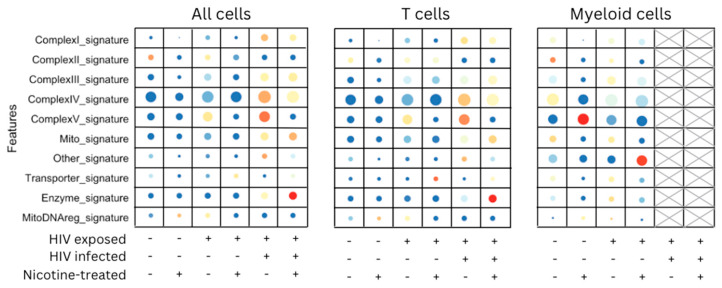
Expression dot plot representation of respiratory gene signatures in all cells, T cells, and myeloid cells under HIV exposure, infection, and nicotine treatment. This figure shows the expression of respiratory gene signatures across three cell populations—all cells, T cells, and myeloid cells—under different experimental conditions, including HIV exposure, HIV infection, and nicotine treatment. The *y*-axis lists respiratory gene signatures, including Complex IV, mitochondrial, transporter, enzyme, and mitochondrial DNA regulation signatures. The *x*-axis represents the experimental conditions, indicated by the presence (+) or absence (−) of HIV exposure, HIV infection, and nicotine treatment. The final two columns for myeloid cells are shaded to indicate that no infected myeloid cells were observed in these conditions. Dot size correlates with the percentage of cells expressing each gene signature, with larger dots indicating higher percentages. The color intensity (blue to red gradient) reflects the average expression level of the gene signature, with red representing higher expression and blue representing lower expression.

Given the heightened immune activation observed, we next examined whether this inflammatory response was linked to increased oxidative stress and mitochondrial dysfunction, which could further exacerbate cellular damage under HIV and nicotine co-exposure. Figure 4 shows the expression of respiratory gene signatures, evaluated across three major cell populations—all cells, T cells, and myeloid cells—under the conditions of HIV exposure, HIV infection, and nicotine treatment. In all cells, the expression of Complex IV signatures and mitochondrial signatures was notably elevated under conditions of HIV infection, as indicated by larger dots and more intense red color, especially in the presence of nicotine. Nicotine-treated HIV-infected cells showed higher expression of these respiratory gene signatures compared to other conditions, suggesting an impact of nicotine on mitochondrial function in the context of HIV infection. In T cells, a similar trend was observed, with increased expression of respiratory genes, particularly in Complex IV and mitochondrial signatures in HIV-infected and nicotine-treated cells. The combination of HIV infection and nicotine led to the highest expression levels of these respiratory pathways. In myeloid cells, the expression of enzyme signatures and mitochondrial signatures was elevated under nicotine treatment, even in the absence of HIV infection. This suggests that nicotine has a distinct impact on myeloid cell metabolic and respiratory functions. HIV-infected myeloid cells showed moderate upregulation of respiratory gene signatures, though the response was less pronounced than in T cells and all cells. Both HIV infection and nicotine treatment influence the expression of respiratory gene signatures, particularly those related to mitochondrial function. The effect is especially pronounced in T cells and in conditions where HIV infection and nicotine co-occur, highlighting the potential for nicotine to exacerbate metabolic stress in HIV-infected cells.

**Figure 5:**
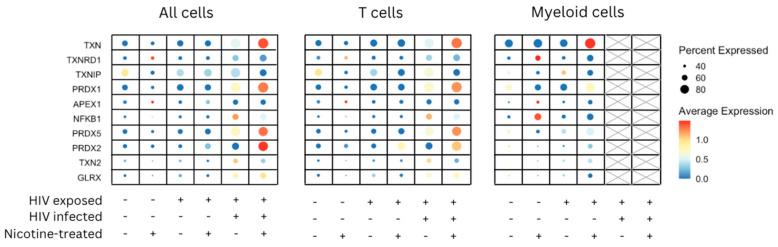
Expression of TXN and related genes in all cells, T cells, and myeloid cells under HIV exposure, infection, and nicotine treatment. This figure illustrates the expression patterns of TXN (thioredoxin) and related oxidative stress genes across three cell populations—all cells, T cells, and myeloid cells—under conditions of HIV exposure, HIV infection, and nicotine treatment. The *y*-axis lists key oxidative stress-related genes, including TXN, TXNRD1, TXNIP, PRDX1, APEX1, NFKB1, PRDX5, PRDX2, TXN2, and GLRX. The *x*-axis denotes combinations of experimental conditions, marked as present (+) or absent (−) for HIV exposure, HIV infection, and nicotine treatment. The final two columns for myeloid cells are shaded to indicate that no infected myeloid cells were observed in these conditions. Dot size represents the percentage of cells expressing each gene. The color intensity (blue to red gradient) reflects the average expression level, with red indicating higher expression and blue indicating lower expression.

As shown in Figure 5, the expression of TXN (thioredoxin) and related oxidative stress genes was evaluated across three cell populations—all cells, T cells, and myeloid cells—under conditions of HIV exposure, HIV infection, and nicotine treatment. In all cells, there was an overall increase in the expression of oxidative stress-related genes, such as TXN, PRDX1, and TXNIP, particularly in conditions where cells were HIV-infected or nicotine-treated. Nicotine treatment, especially when combined with HIV infection, generally amplified the expression of these genes, as indicated by the larger dot sizes and intense red coloration. In T cells, oxidative stress genes like TXN and PRDX1 were upregulated in the presence of both HIV infection and nicotine treatment, with the highest expression levels observed under these combined conditions. However, TXNIP did not follow this trend; instead, its expression appeared slightly reduced under the HIV and nicotine condition compared to HIV infection alone, suggesting that nicotine may not enhance TXNIP expression in HIV-infected T cells. In myeloid cells, the expression of oxidative stress genes was generally lower compared to all cells and T cells, as reflected by smaller dots and lighter color intensity. However, specific genes, including TXNRD1 and PRDX5, showed moderate upregulation in nicotine-treated myeloid cells, particularly in the context of HIV infection. Overall, these findings indicate that both HIV infection and nicotine treatment drive the expression of oxidative stress-related genes, with the most pronounced effects observed in T cells. This suggests that nicotine enhances oxidative stress responses in HIV-infected cells, although the response varies among specific genes, such as TXNIP.

### 3.4. HIV and Nicotine Synergistically Impact Oxidative Phosphorylation and the NLRP3 Inflammasome

Following the observed increase in oxidative stress, we next explored how these disruptions in cellular homeostasis, particularly related to oxidative phosphorylation and inflammasome activation, might synergistically impact key cellular pathways under the combined influence of HIV and nicotine.

**Figure 6:**
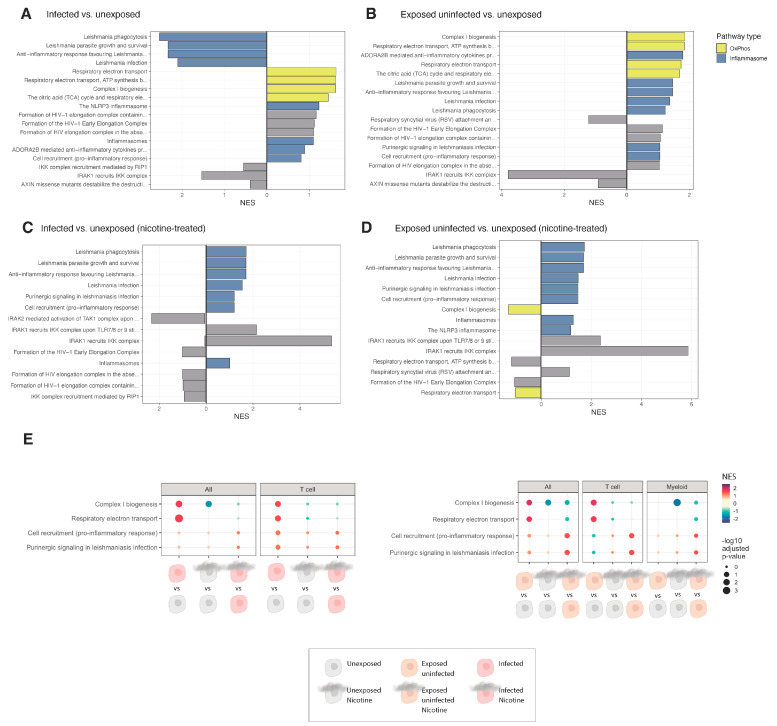
Gene set enrichment analysis (GSEA) for infected vs. unexposed and exposed uninfected vs. unexposed conditions untreated and with nicotine treatment. Gene set enrichment analysis (GSEA) is shown for all cells untreated infected vs. unexposed (**A**) and exposed vs. uninfected (**B**) for and for all cells nicotine-treated infected vs. unexposed (**C**) and nicotine-treated exposed uninfected (**D**). Comparative dot plots are shown (**E**) in cell populations (all, T cells, and myeloid cells) for infected vs. unexposed (**left**) and exposed uninfected vs. unexposed (**right**) conditions. The dot size represents the significance level (*p*-value), and the color intensity indicates the normalized enrichment score (NES). Pathways analyzed include Complex I biogenesis, respiratory electron transport, pro-inflammatory cell recruitment, and purinergic signaling. Color-coded circles represent different experimental conditions: unexposed (gray), exposed uninfected (peach), infected (red), unexposed nicotine (tan), exposed uninfected nicotine (light tan), and infected nicotine (light red).

Gene set enrichment analysis (GSEA) was performed to compare pathway enrichment between infected vs. unexposed and exposed uninfected vs. unexposed conditions under both untreated and nicotine-treated settings, providing insights into the effects of HIV infection and nicotine on various cellular processes. In untreated conditions, GSEA revealed distinct enrichment patterns when comparing infected vs. unexposed cells (Figure 6A) and exposed uninfected vs. unexposed cells (Figure 6B). Notably, pathways related to inflammasome activation, respiratory electron transport, and immune response showed differential enrichment, highlighting the cellular impact of HIV exposure and infection.

Under nicotine treatment, the enrichment patterns differed markedly, with nicotine-treated infected vs. unexposed (Figure 6C) and nicotine-treated exposed uninfected vs. unexposed (Figure 6D) conditions showing increased activation of pro-inflammatory and metabolic pathways. These results suggest that nicotine amplifies pathway enrichment linked to oxidative stress and immune signaling, potentially exacerbating HIV-associated inflammation.

Gene set enrichment analysis (GSEA) (Figure 6E) was performed to compare the enrichment of various pathways across infected and exposed uninfected conditions in different cell populations (all, T cells, and myeloid cells). The comparative dot plot for pathway enrichment analysis reveals significant differences in pathway activation between infected and exposed uninfected conditions. In the infected vs. unexposed condition (left), pathways related to mitochondrial function, specifically Complex I biogenesis and respiratory electron transport, were notably enriched, particularly in T cells. This effect was abrogated with nicotine treatment in both infected and unexposed cells. By contrast, both inflammasome pathways (cell recruitment, pro-inflammatory response, and purinergic signaling in Leishmaniasis infection) showed a notable increase in nicotine-treated infected cells compared to infected and unexposed cells, notably in T cells. In exposed uninfected cells (right), a similar pattern is seen with nicotine treatment in both T cells and myeloid cells, resulting in increased inflammasome pathway enrichment.

**Figure 7:**
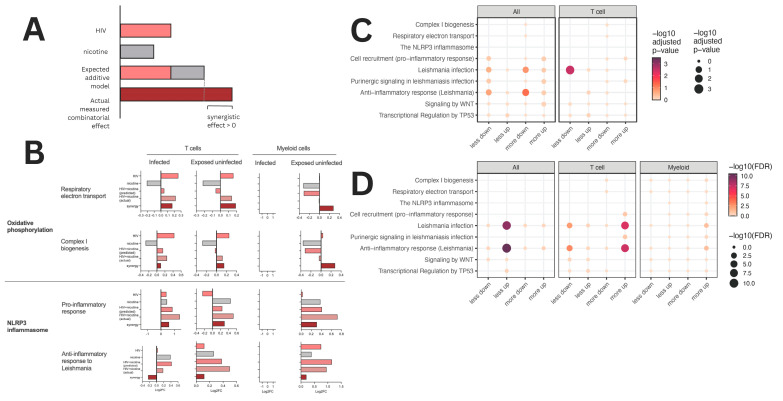
Synergy analysis combined HIV and nicotine exposure on key pathways in T cells and myeloid cells. (**A**) Hypothetical model demonstrating how synergy is calculated. The bar graph compares the expected additive model (gray) to the actual measured combinatorial effect (dark red/maroon) for key biological processes such as Complex I biogenesis, respiratory electron transport, cell recruitment (pro-inflammatory response), and purinergic signaling in Leishmaniasis infection. Synergistic effects occur when the actual measured effect exceeds the predicted additive effect, indicated by a positive synergistic effect (>0). (**B**) Bar graphs represent the log2 fold change (log2FC) in pathway activity for Complex I biogenesis, respiratory electron transport, cell recruitment (pro-inflammatory response), and purinergic signaling in Leishmaniasis infection. Pathway changes are shown for T cells (**left**) and myeloid cells (**right**) under infected (**left**) and exposed uninfected (**right**) conditions. The experimental conditions compared are HIV, nicotine, and combined HIV+ nicotine exposure. The predicted additive effect (gray) is compared with the actual observed effect (dark red/maroon). This synergy analysis highlights the comparison between the predicted additive effect (gray) and the actual observed effect (dark red/maroon). (**C**) Synergy categories displayed using overrepresentation analysis (ORA) for all gene sets in all cells and T cells during infection. Pathway enrichment is shown based on adjusted *p*-values (−log10), with dot sizes indicating the magnitude of significance for each enriched pathway and color indicating the directionality of change. (**D**) Synergy analysis for exposed uninfected cells, showing pathway enrichment across all cells, T cells, and myeloid cells. This analysis visualizes synergy in terms of adjusted *p*-values (−log10 FDR), with dot size reflecting the level of significance and color intensity showing the strength of the synergy effect.

As shown in Figure 7, synergy analysis was performed to assess the combined effects of HIV and nicotine on key biological pathways, comparing the predicted additive effect with the actual observed effect across T cells and myeloid cells. The hypothetical model (Figure 7A) illustrates how the synergy between HIV and nicotine exposure is calculated by comparing the expected additive effects with the measured combinatorial effects. For key biological processes, including Complex I biogenesis, respiratory electron transport, cell recruitment (pro-inflammatory response), and purinergic signaling in Leishmaniasis infection, the predicted additive effects (gray bars) were derived by summing the individual contributions of HIV and nicotine exposure. The actual observed effects (dark red/maroon bars) were then plotted against this additive model (Figure 7B). The bar graphs represent the log2 fold change (log2FC) in pathway activity for Complex I biogenesis, respiratory electron transport, cell recruitment (pro-inflammatory response), and purinergic signaling in Leishmaniasis infection. Pathway changes were analyzed in T cells (left) and myeloid cells (right) under infected and exposed uninfected conditions. Synergy was quantified by determining the deviation of the actual effect from the predicted additive model, with a synergistic effect considered significant when the actual measured effect exceeded the additive prediction. Positive deviations (>0) indicate synergy, where the combined effect of HIV and nicotine is greater than expected from the additive contributions of each factor alone. These synergistic effects are particularly pronounced in pathways related to Complex I biogenesis and cell recruitment (pro-inflammatory response), suggesting that combined HIV and nicotine exposure has an enhanced impact on these key processes compared to either exposure alone.

In infected T cells, pathways such as Complex I biogenesis and respiratory electron transport showed significant positive synergy, with the actual effect of HIV and nicotine being greater than the predicted additive effect. Similarly, cell recruitment (pro-inflammatory response) was significantly upregulated, indicating a synergistic pro-inflammatory response to the combined exposure. In exposed uninfected T cells, synergy was also observed but to a lesser extent. In myeloid cells, purinergic signaling exhibited notable synergy in the infected condition, while other pathways showed less pronounced effects.

Synergy categories are displayed using overrepresentation analysis (ORA) for all gene sets in all cells and T cells during infection (Figure 7C). This analysis highlights the pathways that are significantly enriched under synergistic conditions, with dot size corresponding to the magnitude of pathway significance (adjusted *p*-value) and color indicating the direction of pathway regulation. Pathways such as Complex I biogenesis and respiratory electron transport showed strong enrichment in both T cells and all cells during infection, supporting the observed synergy in these pathways.

The synergy analysis for exposed uninfected cells, across all cells, T cells, and myeloid cells is shown in Figure 7D. This panel visualizes pathway enrichment in terms of adjusted *p*-values (−log10 FDR). While synergy is still present in exposed uninfected cells, it is less pronounced compared to infected cells, with lower overall enrichment across most pathways. These observations suggest that the combination of HIV infection and nicotine exposure leads to significant synergistic effects on key pathways, particularly those involved in mitochondrial function (Complex I biogenesis) and pro-inflammatory responses. These effects are most prominent in T cells and, to a lesser extent, myeloid cells, suggesting that nicotine exacerbates HIV-induced disruptions in cellular metabolism and immune activation.

**Figure 8:**
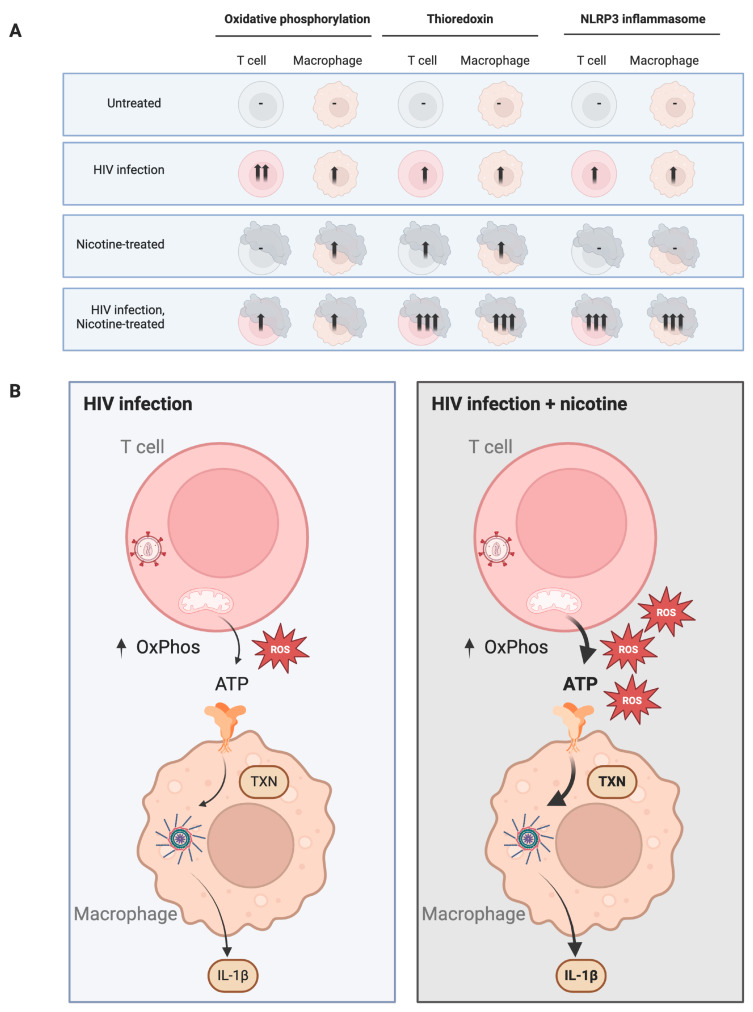
Synergistic effect of nicotine and HIV on oxidative phosphorylation and inflammatory signaling in T cells and macrophages. (**A**) Schematic representation of T cells and macrophages under four conditions: untreated, HIV-infected, nicotine-treated, and HIV-infected with nicotine treatment. The arrows indicate the levels of oxidative phosphorylation (OxPhos) in T cells and inflammasome activation in macrophages across the conditions. HIV infection alone increases OxPhos and ATP release from T cells, leading to inflammasome activation and interleukin-1β (IL-1β) production in macrophages. Nicotine treatment further enhances these effects, leading to higher reactive oxygen species (ROS) production and ATP efflux in T cells, amplifying inflammasome activation and IL-1β secretion in macrophages. (**B**) In the absence of nicotine, HIV infection leads to increased OxPhos and ROS in T cells, resulting in ATP release. This activates the NLRP3 inflammasome in macrophages, producing IL-1β. (C) Nicotine treatment in HIV-infected T cells results in a synergistic increase in OxPhos and ROS, leading to higher ATP efflux and exaggerated inflammasome activation in macrophages. The result is a robust production of pro-inflammatory IL-1β, suggesting that nicotine accelerates HIV-associated inflammation and may play a role in maintaining viral latency through chronic immune activation. Abbreviations: OxPhos, oxidative phosphorylation; ROS, reactive oxygen species; ATP, adenosine triphosphate; IL-1β, interleukin-1β; TXN, transcription; NLRP3, NOD-, LRR-, and pyrin domain-containing protein 3. Created with Biorender.

Figure 8 illustrates the effects of HIV infection and nicotine treatment on oxidative phosphorylation, thioredoxin, and NLRP3 inflammasome activity in T cells and macrophages across four conditions: untreated, HIV infection, nicotine-treated, and HIV infection with nicotine treatment. For oxidative phosphorylation, T cells show a mild increase in activity under HIV infection alone, with further enhancement when HIV is combined with nicotine treatment. In contrast, macrophages do not exhibit significant changes in oxidative phosphorylation under HIV infection alone; however, nicotine treatment, both alone and in combination with HIV, induces a mild increase in this pathway. In the case of thioredoxin, there is a notable increase in T cells under both HIV infection and nicotine treatment, with the strongest upregulation occurring when the two conditions are combined. Similarly, macrophages show moderate elevations in thioredoxin levels with both HIV infection and nicotine, and the combination of HIV and nicotine leads to the highest increase in thioredoxin expression.

For the NLRP3 inflammasome, T cells exhibit an activation in response to HIV infection, which is further amplified when nicotine is added. Macrophages display consistently elevated NLRP3 inflammasome activity across all conditions, with the strongest activation occurring when both HIV and nicotine are present.

## 4. Discussion

The findings of this study demonstrate that HIV infection and nicotine exposure have a synergistic effect on key inflammatory and metabolic pathways in both CD4^+^ T cells and myeloid cells. This synergy is likely driven by the interplay of inflammasome activation, oxidative stress, purinergic signaling, and metabolic reprogramming. Our data provide novel insights into how HIV and nicotine interact at the cellular and molecular levels, with significant implications for the management of PWH, particularly for those who smoke or use nicotine products.

**Inflammasome activation as a central mechanism.** Our results underscore the critical role of the NLRP3 inflammasome in mediating the synergistic effects of HIV and nicotine. The NLRP3 inflammasome was significantly upregulated in both T cells and myeloid cells under conditions of HIV infection, and this effect was further enhanced by nicotine treatment. This dual activation suggests that the inflammasome may serve as a key mediator of the chronic inflammation observed in HIV-infected individuals, especially those exposed to nicotine. Given that inflammasome activation is associated with both HIV persistence and immune dysfunction, these findings suggest that targeting the inflammasome may represent a novel therapeutic approach to reduce HIV reservoirs and limit inflammation-driven tissue damage.

**Oxidative stress and mitochondrial dysfunction.** The observed upregulation of oxidative stress-related genes, including TXN, PRDX1, and APEX1, in HIV-infected and nicotine-treated cells points to a significant role of oxidative stress in driving the synergistic effects of these two factors. Both HIV and nicotine independently increase reactive oxygen species (ROS) production, leading to mitochondrial dysfunction and cellular damage. These stressors likely exacerbate mitochondrial injury, fueling inflammasome activation and promoting viral persistence. This is particularly evident in the upregulation of respiratory gene signatures, such as Complex IV, in both T cells and myeloid cells. These findings suggest that oxidative stress not only contributes to the cellular damage seen in PWH but also amplifies the inflammatory response when combined with nicotine exposure.

**Purinergic signaling and immune modulation.** Purinergic signaling, particularly through the P2X7 receptor, emerged as a critical pathway contributing to the observed synergy. ATP release, a trigger for purinergic receptor activation, was elevated in both HIV-infected and nicotine-treated cells. This ATP binds to P2X7 receptors on immune cells, promoting inflammasome activation and pyroptosis. The synergistic activation of this pathway likely explains the heightened inflammation and immune cell death observed in HIV-infected smokers. These results highlight purinergic signaling as a potential therapeutic target to mitigate the synergistic effects of HIV and nicotine on immune activation and inflammation.

**Metabolic reprogramming and viral persistence.** HIV infection induces significant metabolic reprogramming in infected cells, shifting cellular metabolism towards glycolysis and oxidative phosphorylation. Our results indicate that nicotine further enhances this metabolic reprogramming, particularly by upregulating mitochondrial respiratory pathways. The synergy between HIV and nicotine in altering cellular metabolism may provide a favorable environment for viral replication and persistence while also promoting chronic inflammation. These findings suggest that targeting metabolic pathways in HIV-infected individuals who use nicotine could offer a novel strategy for reducing viral reservoirs and limiting inflammation-driven damage.

**Potential implications of HIV and nicotine synergy for HAND**. Our findings indicate that nicotine enhances HIV-driven inflammation, particularly through mechanisms involving oxidative stress, metabolic reprogramming, and inflammasome activation. Although our study does not directly address CNS integrity, it is plausible that the same pathways contributing to systemic inflammation in our findings may have implications for inflammation in the CNS, especially given nicotine’s known effects on blood–brain barrier integrity and immune activation. The observed synergy between HIV and nicotine in activating inflammasome pathways and purinergic signaling likely amplifies chronic inflammation, which could have broader implications for neurocognitive health in PWH. These insights highlight the need for further investigation into CNS neuroinflammation and microglial involvement, as targeting nicotine’s inflammatory effects—such as through inflammasome inhibition and nicotine cessation—could potentially reduce inflammation and improve outcomes for PWH.

**Inflammasome inhibition as a potential therapy.** Given the central role of the NLRP3 inflammasome in mediating the synergistic effects of HIV and nicotine, NLRP3 inhibitors represent a promising therapeutic strategy for HIV-infected individuals, particularly smokers or nicotine users. By inhibiting inflammasome activation, these therapies could reduce chronic inflammation, limit the expansion of viral reservoirs, and potentially improve overall treatment outcomes. Additionally, nicotine cessation strategies could complement inflammasome inhibition by reducing oxidative stress and further dampening the inflammatory response in HIV-infected patients. These combined approaches may also mitigate comorbidities commonly associated with HIV infection and smoking, such as cardiovascular disease and chronic inflammation.

**Limitations.** While this study provides important insights into the mechanisms of HIV-nicotine synergy, several limitations should be acknowledged. First, human tonsil explants and single-cell RNA sequencing offer valuable high-resolution data but may not fully capture the complexity of in vivo responses. Moreover, while tonsil tissue provides a relevant model for studying HIV infection in lymphoid tissue, it may not fully represent other critical sites of HIV persistence, such as the gut or central nervous system. Further validation of these findings in in vivo models, such as humanized mice, or clinical studies will be necessary to confirm the observed synergy and evaluate its therapeutic implications.

**Future directions—therapeutic exploration.** Further studies are needed to explore the therapeutic potential of targeting the NLRP3 inflammasome in HIV-infected individuals. Future research should investigate the efficacy of NLRP3 inhibitors in reducing viral reservoirs and limiting inflammation in patients who smoke or use nicotine. Additionally, combination therapies that pair anti-retroviral therapy (ART) with inflammasome inhibitors could be explored as a strategy to improve outcomes in HIV treatment.

**Long-term studies on comorbidities.** Given the established link between chronic inflammation and comorbidities in HIV-infected individuals, longitudinal studies are warranted to assess the impact of nicotine use on HIV progression and the development of comorbid conditions, such as cardiovascular disease. Understanding the long-term effects of HIV-nicotine interactions on patient outcomes will be critical for developing comprehensive treatment strategies that address both viral control and the management of comorbidities.

**Combination therapy.** Future studies should also investigate the potential benefits of combining ART with therapies that target inflammation, oxidative stress, or purinergic signaling. By addressing both the viral and inflammatory components of HIV infection, such combination therapies could offer a more effective approach to controlling HIV and improving patient outcomes, particularly in individuals exposed to nicotine.

## 5. Conclusions

This study demonstrates a significant synergy between HIV infection and nicotine exposure, particularly in the activation of inflammatory and metabolic pathways within CD4^+^ T cells and myeloid cells. Our findings reveal that both HIV and nicotine amplify NLRP3 inflammasome activity, oxidative stress, and mitochondrial dysfunction, which may contribute to chronic inflammation, viral persistence, and worsening of comorbid conditions such as cardiovascular disease in PWH.

The data suggest that inflammasome activation plays a central role in mediating this synergy. The upregulation of inflammasome-related genes in T cells and myeloid cells under conditions of HIV infection and nicotine exposure points to the inflammasome as a key driver of immune dysfunction and chronic inflammation in these individuals. This highlights the potential of targeting the inflammasome as a therapeutic strategy for reducing inflammation, limiting viral reservoirs, and improving patient outcomes, especially in those who use nicotine.

Nicotine cessation could also have profound implications for managing HIV, particularly by reducing oxidative stress and mitigating its exacerbation of HIV-induced inflammation. By limiting the harmful interaction between nicotine and HIV, smoking cessation, combined with anti-inflammatory therapies, may provide a comprehensive approach to improving the health of PWH.

While this study provides valuable insights into the molecular mechanisms underlying the HIV-nicotine interaction, it is important to recognize its limitations. Although highly informative, the use of human tonsil explants and single-cell RNA sequencing requires further validation through in vivo models or clinical studies. Future research should focus on confirming these findings in more comprehensive systems, such as humanized mouse models, and exploring the long-term clinical implications of HIV and nicotine interactions.

This work underscores the need for novel therapeutic strategies that target both viral control and the inflammatory processes exacerbated by nicotine use. Combination therapies, integrating anti-retroviral therapy with inflammasome inhibition or oxidative stress reduction, could offer a promising direction for improving the management of PWH, especially for those who smoke or use nicotine. This dual approach has the potential to not only limit viral persistence but also reduce the burden of HIV-associated comorbidities, ultimately enhancing patient care.

## Data Availability

The data supporting the reported results in this study are available upon reasonable request from the corresponding author.
